# Joint Data Transmission and Energy Harvesting for MISO Downlink Transmission Coordination in Wireless IoT Networks

**DOI:** 10.3390/s23083900

**Published:** 2023-04-11

**Authors:** Jain-Shing Liu, Chun-Hung Lin, Yu-Chen Hu, Praveen Kumar Donta

**Affiliations:** 1 Department of Computer Science and Information Engineering, Providence University, Taichung 433, Taiwan; 2 Department of Computer Science and Engineering, National Sun Yat-sen University, Kaohsiung 804, Taiwan; 3 Department of Computer Science and Information Management, Providence University, Taichung 433, Taiwan; 4 Research Unit of Distributed Systems, TU Wien, 1040 Vienna, Austria

**Keywords:** IoT, SWIPT, joint optimization, beamforming, power control, energy harvesting, transmission coordination, deep reinforcement learning

## Abstract

The advent of simultaneous wireless information and power (SWIPT) has been regarded as a promising technique to provide power supplies for an energy sustainable Internet of Things (IoT), which is of paramount importance due to the proliferation of high data communication demands of low-power network devices. In such networks, a multi-antenna base station (BS) in each cell can be utilized to concurrently transmit messages and energies to its intended IoT user equipment (IoT-UE) with a single antenna under a common broadcast frequency band, resulting in a multi-cell multi-input single-output (MISO) interference channel (IC). In this work, we aim to find the trade-off between the spectrum efficiency (SE) and energy harvesting (EH) in SWIPT-enabled networks with MISO ICs. For this, we derive a multi-objective optimization (MOO) formulation to obtain the optimal beamforming pattern (BP) and power splitting ratio (PR), and we propose a fractional programming (FP) model to find the solution. To tackle the nonconvexity of FP, an evolutionary algorithm (EA)-aided quadratic transform technique is proposed, which recasts the nonconvex problem as a sequence of convex problems to be solved iteratively. To further reduce the communication overhead and computational complexity, a distributed multi-agent learning-based approach is proposed that requires only partial observations of the channel state information (CSI). In this approach, each BS is equipped with a double deep Q network (DDQN) to determine the BP and PR for its UE with lower computational complexity based on the observations through a limited information exchange process. Finally, with the simulation experiments, we verify the trade-off between SE and EH, and we demonstrate that, apart from the FP algorithm introduced to provide superior solutions, the proposed DDQN algorithm also shows its performance gain in terms of utility to be up to 1.23-, 1.87-, and 3.45-times larger than the Advantage Actor Critic (A2C), greedy, and random algorithms, respectively, in comparison in the simulated environment.

## 1. Introduction

Given the explosive growth of smart phones and other new applications that result in huge amounts of data transmission apart from the conventional telephone voice service, the massive Internet of Things (IoT) is currently facing significant challenges, such as achieving intelligent implementations [1] and ensuring secure and trustworthy operations [2]. To address these challenges, technologies, such as semi-federated learning [1] and blockchain [2], can be employed. Cellular-based mobile networks will continue to play a crucial role in the development of fifth-generation (5G) and beyond 5G (B5G) wireless communications for IoT, enabling innovative solutions to these challenges.

In such networks, frequency bands are usually reused to mitigate inter-cell interference. Herein, a frequency band shared by all cells is usually considered to have a harmful impact on communication. However, owing to the excessive increase of data traffic, such sharing becomes a possible solution to the problem of scarce radio resources to be used in ultra-dense cellular networks. For this, coordinated multi-point (CoMP) [3] is a promising concept to manage the resulting interference. Specifically, if each BS in the cellular network can perform downlink beamforming [4] for transmitting to its UE appropriately, the intra-cell and inter-cell interference would be mitigated. Given the significant advantage, CoMP is included in the specifications of long term evolution-advanced (LTE-A) [5].

Apart from the interference issue, user equipment (UE) in 5G or B5G is still energy-constrained due to its battery with limited capacity, which is especially true for low-power IoT devices acting as femto UEs within these networks. Despite the slow progress of the battery capacity in recent decades, energy harvesting techniques have emerged to address the crucial issue. As expected, various renewable energy resources could be adopted to refill batteries, such as wind and solar, but their usability is restricted to weather, position, and many other conditions.

In view of these problems, the radio frequency (RF)-based wireless energy transfer (WET) technique would be an alternative that can charge low-power devices over the air, simplify the maintenance procedure, and significantly contribute to the realization of scalable wireless networks [6]. As an extension, WET combined with the wireless network for transmitting information by default results in simultaneous wireless information and power transfer (SWIPT), which enables a UE to harvest energy from the electromagnetic waves in RF from its surroundings while it simultaneously performs information decoding (ID) for the data transmitted from its source [7,8].

### 1.1. Related Work

Based on SWIPT, many related works have been performed. Among them, a pioneering work [9] with a multi-antenna BS transmitting to its UE in downlink was proposed that provides the rate-energy trade-offs for the broadcast SWIPT system involved. In addition, it is shown that each UE can perform ID and EH at the same time with a power splitting (PS) scheme or at different time slots with a time switching (TS) scheme. As an extension of TS, the authors in [10] proposed two new time-splitting schemes, namely time-division mode switching (TDMS) and time-division multiple access (TDMA) for a multi-input single-output (MISO) interference channel (IC). With the possibility of simplifying the receiver design, TS, however, does not actually perform ID and EH simultaneously and would only provide limited exploitation of radio resources [11,12], which motivates the use of PS in this work.

As an example adopting PS, the work [13] resolves a throughput maximization problem subject to energy and temperature constraints at transmitting and receiving nodes, respectively, for a hybrid SWIPT relay system. Extending its viewpoint beyond throughput, the work [14] addresses a fundamental problem to characterize the trade-offs for maximizing energy efficiency (EE) vs. spectrum efficiency (SE) under a point-to-point additive white Gaussian noise (AWGN) channel.

In addition, with respect to orthogonal frequency division multiple access (OFDMA) systems, the related work [15] considered a resource-allocation problem to maximize EE in SWIPT with a PS scheme, and developed fractional programming models and sub-optimal iterative resource allocation algorithms to tackle the nonconvex problems encountered. In [16], with the assumption of using zero-forcing (ZF) beamforming patterns (BPs), the authors aimed to maximize EE under a PS-based MISO downlink system. In [17], a multi-user MISO SWIPT system was considered, and an iterative algorithm was proposed, which is guaranteed to achieve a Karush–Kuhn–Tucker solution for maximizing the EE of this system. Similarly, by focusing on wireless sensor networks, the authors in [18] tackled nonconvex EE optimization problems and proposed sub-optimal iterative algorithms through nonlinear fractional programming and Lagrangian dual decomposition.

Apart from the above, different EH-enabled frameworks can be also found in the literature. For example, the authors in [19] proposed a MOO formulation for a multi-pair two-way relay network to maximize the achievable rates of all *K* UE pairs involved. In that work, by using zero-forcing to null the multi-user interference, the achievable rate of a UE pair can only depend on their own PRs, and the MOO problem can be converted to *K* independent single objective optimization problems. Thus, the trade-off on data rate can be made between UE pairs. However, in our MOO formulation, the inter-cell interference would be involved, and the trade-off between SE and EH in the system is mainly considered rather than the trade-off for the rate between UE pairs in [19].

As another example, a wirelessly powered IoT system was also investigated in [20], wherein sensors harvested energies from the distributed access points (APs) and then transmitted data to the APs with the harvested energies. Although this is different from the SWIPT scenario considered here, how to extend the current work based on the results in [20] giving a higher WET efficiency could be an interesting future work. To see more related works on SWIPT, WET, or both, one may refer to survey papers, such as [21,22].

Despite the various mathematical approaches adopted in the related works that we mentioned, the computational complexity of mobile wireless network has made it impossible to decide all the system parameters required in time. To meet the time constraint, deep learning is a promising data-driven approach that adopts a deep neural network (DNN) to resolve complex nonlinear problems without explicitly formulating complicated mathematical models [23]. Recently, DNN-based learning algorithms have also been developed to resolve different problems in SWIPT-enabled networks as another way to find the solutions in time apart from the analytical-based methods under consideration, which may be not sufficiently time-efficient in usual cases.

As a method based on learning with DNN, the work in [24] proposed a long short-term memory (LSTM) recurrent neural network (RNN)-based mode-switching algorithm to maximize the achievable rate under the energy-causality constraint for its dual mode SWIPT system. In [25], the authors determine the subchannel allocation, power splitting ratio (PR), and transmit power for the SWIPT-based device-to-device (D2D) networks through the deep-reinforcement-learning (DRL)-based algorithm developed therein. For similar D2D SWIPT-based networks, an EE optimization problem was formulated in [26], and the authors adopted exhaustive search (ES) and gradient search (GS), respectively, to obtain the global optimum and local optimum for the formulated nonconvex optimization problem.

In [27], by clustering the antennas into two multiple-input multiple-output (MIMO) subsystems, the authors developed a sub-optimal method and a hybrid DRL method to resolve the combinatorial problem for the full-duplex MIMO system involved, which jointly optimized the antenna clusters and pre-coding matrices for ID and EH so that the weighted sum of their performance metrics can be maximized. In [28], with the multi-user MISO SWIPT-enabled heterogeneous wireless networks as the target, the authors maximized the achievable sum information rate of the femtocells by jointly optimizing BP and PR under the achievable data rate requirements through a multi-agent DDQN algorithm.

### 1.2. The Motivations and Characteristics of This Work

Taking both ID and EH into account, the previous works on SWIPT usually focused on throughput maximization [10,13], EE optimization [15,16,18], or both [14]. As a complement to the above, our work concerns the trade-off between SE and EH in the SWIPT-enabled networks with MISO channels, which is similar to the objective given in [29] for D2D networks without BP decision.

However, the objective considered here is to decide both BP and PR, and our work further reveals that, in addition to the interference management concerned by CoMP, the decisions on BP and PR in SWIPT lead to an overall system utility reflecting both SE and EH with weights to achieve the optimal trade-off subject to the transmit power constraint and the feasible PR constraint. As we know, such a trade-off for the coordinated beamforming in the MISO downlink SWIPT-enabled networks with FP and DRL under the logarithmic nonliner EH model [30,31] is not explicitly explored in the previous works. Specifically, the contributions of this work can be summarized as follows.

- We derive a multi-objective optimization (MOO) formulation to obtain the optimal BP and PR for the MISO downlink SWIPT-enabled wireless networks under the logarithmic nonliner EH model. Then, with a weighted sum approach, we transform this formulation to obtain an objective function for the resulting multiple-ratio FP problem.
- To solve the non-convex FP problem, instead of using the Dinkelbach’s transformation that is usually considered, we develop an evolutionary algorithm (EA)-aided quadratic transform technique that can obtain the desired PR with EA first, and then feed it to an effective iterative algorithm for near-optimal solutions.
- To further reduce the computational complexity while avoiding the collection of global channel state information (CSI), we propose a distributed multi-agent learning-based approach that requires only partial observations of CSI. Specifically, we develop a multi-agent double DQN (DDQN) algorithm for each BS to decide its BP and PR based only on local observations with lower overheads of communication and computation.
- Instead of centralized operations, such as centralized training centralized executing (CTCE) and centralized training distributed executing (CTDE), we adopt a distributed training distributed executing (DTDE) scheme, which makes the offline training and online decision making performed by each single agent or BS distributive and independent and limits the amount of information to be exchanged between neighboring BSs.
- We verify the trade-off between SE and EH with simulations and show that our proposal can outperform the state-of-the-art centralized learning-based algorithm, Advantage Actor Critic (A2C), and baseline approaches, such as greedy and random algorithms. More specifically, it can be seen that, in addition to the introduced FP algorithm to provide superior solutions, the proposed DDQN algorithm can also show its performance gain in terms of utility up to 1.23-, 1.87-, and 3.45-times larger than the A2C, greedy, and random algorithms, respectively, in comparison.

The rest of this paper is structured as follows. In Section 2, we introduce the network, channel model, and problem formulation for this work. Next, we present the EA-aided quadratic transform technique and the FP-based iterative algorithm in Section 3. Then, the limited channel information exchange mechanism is summarized in Section 4, and the distributed multi-agent learning-based DDQN approach is introduced in Section 5. After that, the proposed algorithms are numerically examined in Section 6 to show the trade-offs between SE and EH and their performance differences when compared with other DRL-based algorithms and baseline approaches. Finally, our conclusions are drawn in Section 7.

## 2. System Model and Problem Formulation

### 2.1. Network and Channel Models

**Figure 1:**
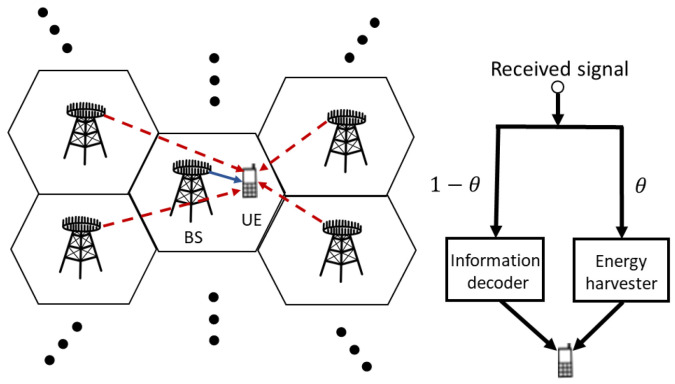
An example of a MISO SWIPT-enabled wireless IoT network model.

As an example shown in Figure 1, the downlink wireless network in question is composed of *L* cells, and, in each cell, there is a BS equipped with $N_{t}$ antennas to transmit to a single-antenna IoT-UE (or UE for short in the sequel). In fact, each cell can support multiple UEs by using orthogonal frequency bands; thus, no intra-cell interference is considered here. However, as noted previously, a frequency band shared by all the cells involved is possible, and inter-cell interference would be concerned. Consequently, when focusing on a frequency band adopted, we can model the channel of this system as multi-cell MISO-IC, in which the received signal at the UE associated with *i*-th BS (or say, direct link *i*) at time *t* can be formulated as

(1) $$y_{i}\left(t\right)=h_{i,i}^{†}\left(t\right)\omega_{i}\left(t\right)x_{i}\left(t\right)+\sum_{j\neq i}h_{i,j}^{†}\omega_{j}\left(t\right)x_{j}\left(t\right)+n_{i}\left(t\right)$$

where $x_{i}$ and $x_{j}$ are the transmitted signals from BS *i* and BS *j*, and their transmit powers $P_{i}$ and $P_{j}$ would satisfy the power constraints $E\{|x_{i}\left|\right\}=P_{i}$ and $E\{|x_{j}\left|\right\}=P_{j}$, respectively. In addition, $h_{i,i}\left(t\right)$ and $\omega_{i}\left(t\right)\in C^{N_{t}\times 1}$ denote, respectively, the downlink channel vector and BP of BS *i* toward its UE during time slot *t*, while $h_{i,j}\left(t\right)$ and $\omega_{j}\left(t\right)\in C^{N_{t}\times 1}$ represent the cross-link channel between UE *i* and BS *j*, and BP of BS *j*, respectively. Finally, $n_{i}\in \mathrm{CN}\left(0,\sigma^{2}\right)$ is the overall noise at UE *i*.

In the above, we assume that $N_{t}$ antennas of each BS are arranged as a uniform linear array (ULA). In addition, similar to [9,11,25,26], we consider that each UE *i* with PS on the received signal $y_{i}\left(t\right)$ can simultaneously perform ID and EH as shown in Figure 1. Specifically, with $\theta_{i}\left(t\right)\in \left(0,1\right)$ to denote the PR adopted by UE *i* at time *t*, the instantaneous signal to interference and noise ratio (SINR) for ID can be formulated as [32]:

(2) $$Y_{i}\left(t\right)=\left(1-\theta_{i}\left(t\right)\right)\frac{|h_{i,i}^{†}\left(t\right)\omega_{i}{\left(t\right)|}^{2}}{\sum_{j\neq i}{\left|h_{i,j}^{†}\omega_{j}\left(t\right)\right|}^{2}+\sigma^{2}}$$

Consequently, the achievable data rate of UE *i* would be

(3) $$C_{i}^{d}\left(t\right)=\log\left(1+Y_{i}\left(t\right)\right)$$

On the other hand, the signal split for EH can be denoted by

(4) $$y_{i}^{EH}\left(t\right)\;=\;\sqrt{\theta_{i}\left(t\right)}(h_{i,i}^{†}\left(t\right)\omega_{i}\left(t\right)x_{i}\left(t\right)+\sum_{j\neq i}h_{i,j}^{†}\omega_{j}\left(t\right)x_{j}\left(t\right)+n_{i}\left(t\right))$$

Given this, the conventional works, such as [9,11,25,33,34], usually convert the received signal $y_{i}^{EH}\left(t\right)$ into the DC power with a linear function. However, a nonlinear function for the energy conversion would be more practical, and the previous works, such as [30,31], adopted the logarithmic nonliner EH model for the *i*th IoT device on the *j*th sub-carrier as follows:

(5) $$e_{i}^{h}\left(t\right)=a_{i}\log(1+b_{i}{\left|h_{i,j}\right|}^{2}p_{i,j})$$

where $a_{i}$ and $b_{i}$ are the nonlinear model parameters, and $p_{i,j}$ is the transmission power for the *i*th device on the *j*th sub-carrier with the assumption that the noise power is negligible [30,31]. Following the model without its assumption, this work considers $h_{i,j}$ as the channel between UE *i* and BS *j* on the same frequency band as shown previously, and in terms of these notations, the energy harvested through the split part for EH would be denoted by

(6) $$E_{i}^{h}\left(t\right)=\theta_{i}\left(t\right)(a_{i}\log(1+b_{i}(\sum_{\forall j}|h_{i,j}^{†}\omega_{j}\left(t\right){|}^{2}+\sigma^{2})))$$

### 2.2. Multi-Objective Optimization

Based on the model with SWIPT, our aim is to jointly optimize BP and PR to obtain the maximal SE and EH simultaneously subject to the transmit power constraint and the feasible PR constraint in the MISO downlink network, which can be classified as a MOO problem. As noted in [35], MOO refers to as a type of optimization that involves multiple objective functions to be optimized simultaneously. In general, a nontrivial MOO problem does not have a single solution to concurrently optimize each of the objective functions involved. In such a general case known as conflicting, a Pareto optimization solution is usually pursued wherein none of the objective functions can be improved without degrading some of the other objectives in value. More specifically, it can be defined as a maximization problem as follows [35]:

**Definition 1.**  *Given $f_{i}\in C\to R,1\leq i\leq I$, and X being the feasible set of constraints, a multi-objective optimization problem can be represented by*

(7) $$\begin{array}{c} \begin{array}{cc} \overset{{}_{\mathrm{maximize}}}{{}_{x}} & f\left(x\right)=(f_{1}\left(x\right),\ldots ,f_{I}\left(x\right))\\ \mathrm{subject to} & x\in X\; \end{array} \end{array}$$

For such an optimization problem, there may be feasible solutions to be obtained, which are denoted by Y=f(x). In particular, these solutions are considered efficient if they satisfy the following definition (as Definition 2.1 of [35]):

**Definition 2.**  *A point x∈X is called Pareto optimal if there does not exist other $x^{\prime }\in X$ such that $f\left(x^{\prime }\right)⪰f\left(x\right)$, where ⪰ denotes the component-wise inequality.*

In some cases, it would be easier to find the solutions that are called weakly Pareto optimal for the problems to be relaxed. Consequently, the following definition (as in Definition 2.24 of [35]) could be considered more often:

**Definition 3.**  *A point x∈X is called weakly Pareto optimal if there does not exist other $x^{\prime }\in X$ such that $f\left(x^{\prime }\right)≻f\left(x\right)$, where ≻ denotes the strict component-wise inequality.*

Given these definitions, relevant works could aim to find (weakly) Pareto optimal points or solutions of their MOO problems. Similarly, for our problem, the weighted sum method exemplifying a simple scalarization technique as typically adopted is considered here and can collapse the vector objective into a single-objective sum as

(8) $$\begin{array}{cc} \overset{{}_{\mathrm{maximize}}}{{}_{{}_{x\in X}}} & \sum_{i=1}^{I}W_{i}f_{i}\left(x\right) \end{array}$$

where each $W_{i},1\leq i\leq I$ denotes a non-negative real-valued weight for function $f_{i}$. In particular, as noted in Proposition 3.9 of [35], the optimal solution of problem (8) and the Pareto optimal points of problem (7) have the following relationship:

**Proposition 1.**  If $x^{*}$ is an optimal solution of problem (8), then $x^{*}$ is weakly efficient for the MOO problem (7).

### 2.3. Problem Formulation

As shown above, the MOO problem in question is to simultaneously maximize SE and EH from *L* cells in the MISO downlink network by jointly optimizing BP $\left\{\omega_{i}\right\}$ and PR $\left\{\theta_{i}\right\},\forall i$, subject to the transmit power constraint, and the feasible constraint for PR. Specifically, to simplify our representation in the following, this MOO problem is formulated without the time index *t* as follows:

(9) $$\begin{array}{c} \begin{array}{cccc} \overset{{}_{\max}}{{}_{\omega_{i},\theta_{i}\forall i}} & \left(C^{d}\left(\Omega ,\theta \right),E^{h}\left(\Omega ,\theta \right)\right) & & \left(a\right)\\ \mathrm{subject}\mathrm{to} & P_{min}\leq \left|\right|\omega_{i}{\left|\right|}^{2}\leq P_{max}, & \forall i & \left(b\right)\\ & 0\leq \theta_{i}\leq 1, & \forall i & \left(c\right) \end{array} \end{array}$$

where the sum of the data rates and that of the harvested energies, i.e., $C^{d}\left(\Omega ,\theta \right)=\sum_{\forall i}C_{i}^{d}$ and $E^{h}\left(\Omega ,\theta \right)=\sum_{\forall i}E_{i}^{h}$ in (9a), are the two objective functions to be maximized with $\Omega =\left\{\omega_{1},\omega_{2},\ldots ,\omega_{L}\right\}$ and $\theta =\left\{\theta_{1},\theta_{2},\ldots ,\theta_{L}\right\}$. Apart from the above, it is worth noting that, due to the MOO formulation to maximize the metrics concurrently, no minimum data rate and harvested power are required to be the constraints for each cell involved. Instead, it applies (9b) to enforce that the transmit power $P_{i}$ should be given within the range between the minimum transmit power $P_{min}$ and the maximum transmit power $P_{max}$ and uses (9c) to ensure $\theta_{i}$ is a nonnegative real number that is no larger than 1.

By means of the weighted sum approach (8) introduced in Section 2.2, which can produce a single-objective sum for the vector objective, the objective of this MOO problem (9) is represented here by

(10) $$U\left(\Omega ,\theta \right)=W\frac{C^{d}\left(\Omega ,\theta \right)}{\bar{C^{d}}}+\left(1-W\right)\frac{E^{h}\left(\Omega ,\theta \right)}{\bar{E^{h}}}$$

where $W=W_{i}\in \left(0,1\right],\forall i$ represents the weight for all the cells or BSs. Clearly, it determines the importance between SE and EH in the system objective. In addition, $\bar{C^{d}}$ and $\bar{E^{h}}$ denote the estimated maximal values of $C^{d}\left(\Omega ,\theta \right)$ and $E^{h}\left(\Omega ,\theta \right)$, respectively, which could be obtained from the initial phase with random Ω and θ, which is performed many times in our simulation. These values are utilized here to normalize the two metrics (the data rate and the harvested energy) lying in very different numerical scales. Thus, even with only their estimations, the resulting utility could still be fine-tuned by adjusting *W* and 1−W in (10) to meet the specific balance requirements from users on these metrics if required.

## 3. Fractional Programming-Based Approach

In this work, instead of using the classic Dinkelbach’s transformation [36] that is typically adopted for single-ratio FP problems, we adopt the quadratic transform technique developed in [37] for multi-ratio FP problems. Specifically, for the first objective in (10) aiming at SE, which involves SINR with fractional terms in the logarithm function, we adopt a Lagrangian dual reformulation with a set of dual or auxiliary variables $\gamma =\left\{\gamma_{1},\gamma_{2},\ldots ,\gamma_{L}\right\}$. According to Proposition 2 of [37], the SE objective can be reformulated as

(11) $$\;U^{SE}\left(\Omega ,\theta \right)\;=\;\sum_{i}\left(W_{1}\log\left(1+\left(1-\theta_{i}\right)\gamma_{i}\right)-W_{1}\left(1-\theta_{i}\right)\gamma_{i}+\frac{W_{1}\left(1+\left(1-\theta_{i}\right)\gamma_{i}\right){\left|h_{i,i}^{†}\omega_{i}\right|}^{2}}{\sum_{j}{\left|h_{i,j}^{†}\omega_{j}\right|}^{2}+\sigma^{2}}\right)$$

where $W_{1}=W/\bar{C^{d}}$, and this ignores the time index *t* as noted previously. Then, by taking partial differentiation with respect to $\gamma_{i}$ and leading the result to zero, i.e., $\frac{\partial U^{SE}}{\partial \gamma_{i}}=0$, we can obtain the optimal dual variable for SE as

(12) $$\gamma_{i}=\left(\frac{|h_{i,i}^{†}\omega_{i}{|}^{2}}{\sum_{j\neq i}{\left|h_{i,j}^{†}\omega_{j}\right|}^{2}+\sigma^{2}}\right)/\left(1-\theta_{i}\right)$$

On the other hand, the EH objective in (10) can be also denoted by $W_{2}\left(\log\left(1+b_{i}(\sum_{\forall j}|h_{i,j}^{†}\omega_{j}\left(t\right){|}^{2}+\sigma^{2})\right)\right)$ with $W_{2}=\left(1-W\right)a_{i}\theta_{i}/\bar{E^{h}}$. Then, as the SE counterpart, we can conduct a set of dual variables $\alpha =\left\{\alpha_{1},\alpha_{2},\ldots ,\alpha_{L}\right\}$, and apply the transform similar to that in Proposition 2 of [37] to reformulate the EH objective as

(13) $$U^{eh}\left(\Omega ,\theta \right)\;=\;\sum_{i}\left(W_{2}\log\left(1+\alpha_{i}\right)-W_{2}\alpha_{i}+\frac{W_{2}\left(1+\alpha_{i}\right)b_{i}(\sum_{j}|h_{i,j}^{†}\omega_{j}{|}^{2}+\sigma^{2})}{b_{i}(\sum_{j}|h_{i,j}^{†}\omega_{j}{|}^{2}+\sigma^{2})+1}\right)$$

Similarly, by $\frac{\partial U^{eh}}{\partial \alpha_{i}}=0$, the optimal dual variable for EH with respect to *i* can be given by

(14) $$\alpha_{i}=b_{i}(\sum_{j}|h_{i,j}^{†}\omega_{j}{|}^{2}+\sigma^{2})$$

However, for the consistency with $U^{SE}$, we adopt $U^{EH}\approx U^{eh}$ to have the same denominator in the last term of $U^{SE}$ as follows:

(15) $$U^{EH}\left(\Omega ,\theta \right)\;=\;\sum_{i}\left(W_{2}\log\left(1+\alpha_{i}\right)-W_{2}\alpha_{i}+\frac{W_{2}\left(1+\alpha_{i}\right)b_{i}(\sum_{j}|h_{i,j}^{†}\omega_{j}{|}^{2}+\sigma^{2})-1}{b_{i}(\sum_{j}|h_{i,j}^{†}\omega_{j}{|}^{2}+\sigma^{2})}\right)$$

Finally, (11) and (15) can be combined, leading to the new overall utility as

(16) $$\bar{U}\left(\Omega ,\theta \right)=\frac{\left(W_{1}\left(1+\left(1-\theta_{i}\right)\gamma_{i}\right)+W_{2}\left(1+\alpha_{i}\right)\right){\left|h_{ii}^{†}\omega_{i}\right|}^{2}}{\sum_{j}{\left|h_{i,j}^{†}\omega_{j}\right|}^{2}+\sigma^{2}}+C$$

where $C=C_{1}+C_{2}+C_{3}$ is the independent part that does not directly relate to the transmit signal $h_{i,i}^{†}\omega_{i}$ in the numerator of (20), including

(17) $$\begin{array}{ccc} C_{1} & = & W_{1}\log\left(1+\left(1-\theta_{i}\right)\gamma_{i}\right)-W_{1}\left(1-\theta_{i}\right)\gamma_{i} \end{array}$$

(18) $$\begin{array}{ccc} C_{2} & = & {\hat{W}}_{2}\log\left(1+\alpha_{i}\right)-W_{2}^{\prime }\alpha_{i} \end{array}$$

(19) $$\begin{array}{ccc} C_{3} & = & \frac{W_{2}\left(1+\alpha_{i}\right)\left(\sum_{j\neq i}{\left|h_{i,j}^{†}\omega_{j}\right|}^{2}+\sigma^{2}-1/b\right)}{\sum_{j}{\left|h_{i,j}^{†}\omega_{j}\right|}^{2}+\sigma^{2}} \end{array}$$

where ${\hat{W}}_{2}=W_{2}/b$, and $b=b_{i},\forall i$. However, with the signal from BS *i* to its receiver, i.e., $h_{i,i}^{†}\omega_{i}$, as the major part to be optimized, this formulation would lead to a BP focusing on the data rate to its receiver while ignoring the interference powers from the others to be harvested. To resolve this problem, the numerator part of $C_{3}$ is modified to account for the powers transmitted from BS *i* to the others as $W_{2}\left(1+\alpha_{i}\right)\left(\sum_{j\neq i}{\left|h_{j,i}^{†}\omega_{i}\right|}^{2}+\sigma^{2}-1\right)$ rather than the powers received from the others that cannot be controlled by BS *i* itself in the original form. Consequently, the overall utility function is modified as

(20) $$\hat{U}\left(\Omega ,\theta \right)=\frac{W_{1}\left(1+\left(1-\theta_{i}\right)\gamma_{i}\right){\left|h_{ii}^{t}\omega_{i}\right|}^{2}+W_{2}\left(1+\alpha_{i}\right)\left(\sum_{j\neq i}{\left|h_{j,i}^{†}\omega_{i}\right|}^{2}+\sigma^{2}-1\right)}{\sum_{j}{\left|h_{i,j}^{†}\omega_{j}\right|}^{2}+\sigma^{2}}+\hat{C}$$

where $\hat{C}=C_{1}+C_{2}-{\hat{W}}_{2}\left(1+\alpha_{i}\right)/(\sum_{j}|h_{i,j}^{†}\omega_{j}{|}^{2}+\sigma^{2})$ is not directly related to the transmit signals, $h_{j,i}^{†}\omega_{i},\forall j$, of BS *i*. Then, by using the quadratic transform in the multidimensional and complex case in Theorem 2 of [37] on the UE part and the SE part of (20) without $\hat{C}$, respectively, we have the system objective as

(21) Q^(Ω,θ)=∑i=1L(2(W1(1+(1−θi)γi)Reωi†hi,i†yi+W2(1+αi)∑jReωi†hj,i†yi)−yi†σ2I+∑j≠ihi,jωjωj†hi,j†yi)

where $y_{i}$ is the dual variable in this case. Essentially, the objective is developed to facilitate solving this problem iteratively. That is, when Ω and the other variables are fixed, the optimal $y_{i}$ can be found by solving the first-order optimality, i.e., $\frac{\partial \hat{Q}}{\partial y_{i}}=0$, and the result is

(22) $$\;y_{i}\;=\;{\left(\sigma^{2}\hat{I}+\sum_{j}h_{i,j}\omega_{j}\omega_{j}^{†}h_{i,j}^{†}\right)}^{-1}\left(\sqrt{W_{1}\left(1+\left(1-\theta_{i}\right)\gamma_{i}\right)}h_{i,i}\omega_{i}+\sqrt{W_{2}\left(1+\alpha_{i}\right)}\sum_{j}h_{j,i}\omega_{i}\right)$$

Similarly, the optimal $\omega_{i}$ can be obtained by

(23) $$\omega_{i}={\left(\eta_{i}\hat{I}+\sum_{j}h_{j,i}^{†}y_{j}y_{j}^{†}h_{j,i}\right)}^{-1}\left(\sqrt{W_{1}\left(1+\left(1-\theta_{i}\right)\gamma_{i}\right)}h_{i,i}^{†}y_{i}+\sqrt{W_{2}\left(1+\alpha_{i}\right)}\sum_{j}h_{j,i}^{†}y_{i}\right)$$

In the above, $\eta_{i}$ is the dual variable introduced for the power constraint, and its optimal value can be denoted by

(24) $$\eta_{i}=\min\left\{\eta_{i}\geq 0:P_{min}\leq \left|\right|\omega_{i}\left(\eta_{i}\right){\left|\right|}^{2}\leq P_{max}\right\}$$

which can be efficiently determined by means of a bisection search algorithm.

Apart from the above, it can also be seen that the formulations for $\gamma_{i},\alpha_{i},y_{i}$, and $\omega_{i}$ explored so far all involve $\theta_{i}$. In fact, $\theta_{i}$ is highly coupled among these formulas, and could not be easily resolved through them. For the resulting non-convexity, we resort to evolutionary algorithms (EAs) to find its value to approach the overall optimal solution. Specifically, we develop a simulated annealing (SA) algorithm for this aim as was implemented in [38]. Given this, the FP algorithm to maximize the objective (21) is summarized in Algorithm 1.   
**Algorithm 1** EA-aided FP algorithm.1:Provide $\ell_{m}$, $\ell_{\eta }$, δ, $P_{min}$, and $P_{max}$;2:Initialize θ,γ,α,y,ω, $\eta_{min}$, $\eta_{max}$, and set $c_{1}=0$;3:**repeat**4:   Obtain θ with SA;5:   Update $y_{i},\forall i$, with (22) while fixing γ, α, ω, and θ;6:   **for** each BS or direct link *i* **do**7:     Set $c_{2}=0$, $\bar{P_{min}}=P_{min}$, and $\bar{P_{max}}=P_{max}$;8:     **while** $|\bar{P_{max}}-\bar{P_{min}}|\;>\delta$ and $c_{2}\leq \ell_{\eta }$ **do**9:        Obtain $\omega_{min}$ and $\omega_{max}$ with $\eta_{min}$ and $\eta_{max}$, respectively, through (23) while fixing γ, α, y, and θ;10:        Let $\bar{P_{min}}=\left|\right|\omega_{min}{\left|\right|}^{2}$ and $\bar{P_{max}}=\left|\right|\omega_{max}{\left|\right|}^{2}$;11:        Let $\eta_{mid}=\frac{\eta_{min}+\eta_{max}}{2}$;12:        Obtain $\omega_{mid}$ with $\eta_{mid}$ through (23) while fixing γ, α, y, and θ;13:        Let $\bar{P_{mid}}=\left|\right|\omega_{mid}{\left|\right|}^{2}$;14:        **if** $\bar{P_{mid}}>\bar{P_{max}}$ **then**15:          Let $\eta_{min}=\eta_{mid}$;16:        **else**17:          Let $\eta_{max}=\eta_{mid}$;18:        **end if**19:        $c_{2}=c_{2}+1$;20:     **end while**21:     Update $\omega_{i}$ as $\omega_{mid}$;22:   **end for**23:   Update $\gamma_{i}\;\text{and}\;\alpha_{i},\forall i,$ with (12) and (14), respectively, while fixing ω and θ;24:   $c_{1}=c_{1}+1$;25:**until** convergence or $c_{1}>\ell_{m}$

Note that, although SA is well defined in the literature, our work still requires the FP iterative update procedure with certain modifications to be the fitness function for SA. Specifically, by regarding $\theta_{i}$ as the variable to be updated by the SA algorithm with the same FP iterative update process on the others (i.e., $\gamma_{i}$, $\alpha_{i}$, $y_{i}$, and $\omega_{i}$), the resulting iterative-based fitness function, for example, the SA-Fitness function, can output the desired $\theta_{i}$ with a very limited number of iterations. More explicitly, let ${\hat{\ell }}_{m}$ be the iteration number of the outer loop and ${\hat{\ell }}_{\eta }$ be that of the inner loop in the SA-Fitness function.

Through our experiments, ${\hat{\ell }}_{m}=1$ and ${\hat{\ell }}_{\eta }=100$ can be found to quickly estimate $\theta_{i}$, and we can then input the obtained $\theta_{i}$ into the EA-aided FP algorithm. Given this, our simulations in Section 6.2 confirm the effectiveness of the FP algorithm to provide the system performance metrics outperforming those from the learning-based algorithms and the baseline approaches in comparison.

In summary, the FP-based approach is developed to be an iterative algorithm, which involves (1) obtaining $\theta_{i}$ through SA, (2) updating $y_{i}$ with (22), (3) updating $\omega_{i}$ with (23), (4) updating $\gamma_{i}$ with (12), (5) updating $\alpha_{i}$ with (14), and (6) finding $\eta_{i}$ with the bisection search under the limit of $\ell_{\eta }$ iterations, while fixing the other variables in each step within the total number of $\ell_{m}$ iterations. In the iterative updates, the inverse operation is required to find, e.g., $\omega_{i}$, with the time complexity $O(LN_{t}^{3})$, and the number of $\ell_{\eta }$ bisection-search iterations is also required to find η. Further, to obtain $\theta_{i}$, SA implemented in [38] would expand $O(I_{c}N_{g})$ steps to perform the cost evaluation, where $I_{c}$ is the number of individuals to evaluate in a chain for every generation of SA, and $N_{g}$ is the number of generations to evolve. Given this, its total time complexity would be $O(\ell_{m}I_{c}N_{g}\ell_{\eta }LN_{t}^{3}$).

## 4. Limited Channel Information Exchange

In the networks with MISO downlink channels, a practical approach that is frequently adopted is using BSs to collect the channel information. That is, a BS will obtain the channel measurement through the feedback from UE. To this end, there would exist a backhaul network to carry the global instantaneous CSI collected and transmit it to the central controller for global optimization. However, the signal overhead can be huge, which makes a centralized optimization approach infeasible in a highly dynamic environment.

To alleviate the problem in a practical way, our distributed learning-based approach will utilize only the basic operations of BS to exchange information with other BSs through predefined interfaces, such as X2 in LTE, resulting in a considerably lower signal overhead than that of the backhaul network for centralized optimization. Given this, we consider that each direct link *k* has two limited sets, namely interferers and interfered neighbors, similar to those in [39,40]. Specifically, we limit the number of neighbor *U* of link *k* with the dynamic thresholds $\varphi_{I_{k}}$ and $\varphi_{O_{k}}$ in the following two limited sets:

(25) $$\begin{array}{ccc} & & I_{k}=\left\{j\neq k:\;|h_{k,j}^{†}\omega_{j}{|}^{2}\geq \varphi_{I_{k}}\right\}\\ & & O_{k}=\left\{i\neq k:\;|h_{i,k}^{†}\omega_{k}{|}^{2}\geq \varphi_{O_{k}}\right\} \end{array}$$

where the two thresholds lead to $|I_{k}|=U$ and $|O_{k}|=U$, respectively.

Now, with a control channel to return the feedback, BS *k* at current time *t* can obtain the channel gain $|h_{k,k}^{†}\left(t\right)\omega_{k}{\left(t-1\right)|}^{2}$ and the interference-plus-noise $\sum_{j\neq k}{\left|h_{k,j}^{†}\left(t\right)\omega_{j}\left(t-1\right)\right|}^{2}+\sigma^{2}$ through $\omega_{j}\left(t-1\right),\forall j$, measured by UE *k* at the previous time t−1 as well as the current channel vector $h_{k,j}\left(t\right),\forall j$. Similarly, BS *k* can send its own measurements to its interferers $j\in I_{k}$ and interfered neighbors $i\in O_{k}$ and receive the measurements from the two sets of neighbors as conducted in the previous works. The information for these measurements locally exchanged among the neighbors would then be utilized in the following multi-agent DDQN algorithm, which details the measurements to be adopted therein.

## 5. Learning-Based Approach

In addition to the indicated signal overhead, an optimization-based approach could also have a computational complexity for solving the MOO problem that is non-deterministic polynomial time (NP) in general. Although the FP-based algorithm could be computationally-efficient with the iterative update procedure proposed, to further reduce the signal overhead as well as the computational complexity, we develop a deep-reinforcement-learning-based algorithm to track the fast time-varying channels involved and provide its solutions in a time that could hardly be achieved by using the traditional optimization methods. Specifically, a multi-agent DDQN algorithm is introduced next to make each single agent or BS share only limited information exchanged among its neighbors, effectively reducing the overhead and complexity as mentioned.

### 5.1. Overview of DDQN

In principle, a reinforcement-learning (RL) algorithm has one or more agents to interact with the environment and to take actions based on certain strategies so that the accumulated reward can be maximized in the long term. The interaction between agent(s) and the environment is usually modeled as a Markov decision process (MDP). The well-known Q-learning algorithm is a MDP-based approach, represented here by a four-tuple structure <S,A,R,P>, where S is the set of states, A is the set of discrete actions, R is the reward, and *P* is the transition probability. Specifically, given *r* as the instant reward and ν∈[0,1) as the discount factor, the cumulative discounted reward can be obtained by

(26) $$R_{t}=\sum_{\tau =0}^{\infty }\nu^{\tau }r\left(t+\tau +1\right)$$

Given this, the Q-function associated with a policy π is the expected reward defined by

(27) $$Q_{\pi }\left(s,a\right)=E_{\pi }\left\{R_{t}|s_{t}=s,a_{t}=a\right\}$$

where a∈A is an action taken in state s∈S in time *t*, and the optimal policy $\pi^{*}\left(a|s\right)$ is a mapping from states to actions that maximizes the long-term cumulative discount reward. Then, through the concept of a one-step Markov process, it considers $R\left(s,a\right)=E_{\pi }\left\{r_{t+1}|s_{t}=s,a_{t}=a\right\}$ as the expected instant reward resulting from taking action *a* in state *s* and the transition probability $P_{ss^{\prime }}^{a}=\mathrm{Pr}\left(s_{t+1}=s^{\prime }|s_{t}=s,a_{t}=a\right)$. Given this, the Q-function can be iteratively obtained by using the Bellman Equation [41]

(28) $$Q_{\pi }\left(s,a\right)=R\left(s,a\right)+\nu \sum_{s^{\prime }\in S}P_{ss^{\prime }}^{a}\left(\sum_{a^{\prime }\in A}\pi \left(s^{\prime },a^{\prime }\right)Q_{\pi }\left(s^{\prime },a^{\prime }\right)\right)$$

Accordingly, to find the optimal policy $\pi^{*}$, the Q-learning algorithm is conducted to find the optimal action *a* in state *s*.Through the Bellman equation shown in above, the optimal Q-function associated with the optimal policy $\pi^{*}\left(a|s\right)$ can be represented by

(29) $$Q^{\pi^{*}}\left(s,a\right)=R\left(s,a\right)+\nu \sum_{s^{\prime }\in S}P_{ss^{\prime }}^{a}\max_{a^{\prime }}Q^{\pi^{*}}\left(s^{\prime },a^{\prime }\right)$$

Clearly, to obtain the optimal results, all state–action pairs should be stored in a place, namely the Q-table, in this algorithm, whose dimensions are |S|×|A|, and this could be huge for a general application. Thus, the primitive Q-learning algorithm may be useful only when the state–action space is relatively small, which seriously limits its applicability. Fortunately, by replacing the Q-table with a neural network to find the optimum, the deep-learning algorithm that results, namely DQN, can significantly reduce the overhead, where the Q-function is denoted by Q(s,a|ϕ) with ϕ to denote the weight of DNN. Now, with the learning rate α∈(0,1], the Q-value can be updated by

(30) $$Q\left(s,a|\phi \right)=\left(1-\alpha \right)Q\left(s,a|\phi \right)+\alpha (r+\nu \max_{a^{\prime }}Q\left(s^{\prime },a^{\prime }|\phi \right))$$

The weights of DNN, however, can diverge due to a high correlation between the actions and states that exist, and the algorithm is not guaranteed to converge on the optimal value function. To resolve this problem, apart from the introduced DNN, $Q_{train}$, another DNN, $Q_{target}$, is added to keep a copy of DNN and use it for the Q-value update in the Bellman equation. The two different DNNs have different Q-functions, $Q(s,a|\phi_{1})$ and $Q(s,a|\phi_{2})$. The loss between them can then be defined by

(31) $$L=\sum_{\langle s,a,r,s^{\prime }\rangle }(Q_{target}^{DQN}-Q\left(s,a|\phi_{1}\right))$$

where $Q_{target}^{DQN}=r^{\prime }+\nu \max_{a^{\prime }}Q\left(s^{\prime },a^{\prime }|\phi_{2}\right)$, and minimizing this loss would lead to the optimal solution. Now, even given the loss function, the DQN algorithm may still significantly diverge by overestimating the value of $Q_{target}$. The overestimating problem with respect to the deep deterministic policy gradient (DDPG) algorithm was also indicated in [42,43]. Additionally, DDPG has the potential to become unstable, and its performance may rely on finding the appropriate hyperparameters for a given problem [42]. Therefore, it is currently not being considered in this work.

Instead, a variant approach, namely double DQN (DDQN) as proposed in [44], is considered to select the actions and evaluate the Q-values separately. In particular, unlike DQN directly using the maximum Q-value for the target network, DDQN selects the action from the train network that yields the maximum Q-value, i.e., ${\arg\max}_{a^{\prime }}Q\left(s^{\prime },a^{\prime }|\phi_{1}\right)$ and then identifies the Q-value in the target network by means of the selected action, i.e., $Q(s^{\prime },{\arg\max}_{a^{\prime }}Q\left(s^{\prime },a^{\prime }|\phi_{1}\right)|\phi_{2})$. Finally, the Q-value for $Q_{target}$ in DDQN can be obtained by

(32) $$Q_{target}^{DDQN}=r^{\prime }+\nu Q(s^{\prime },\underset{a^{\prime }}{\arg\max}Q\left(s^{\prime },a^{\prime }|\phi_{1}\right)|\phi_{2})$$

Apart from the potential to resolve the overestimating problem, DDQN was also shown to obtain the best results through certain datasets for training [45] and the lowest cost for the dynamic context delivery when compared with the others [46]. In addition, as shown in [44], the lower bound on the absolute error of DDQN estimate is zero. Given these good properties, we develop, in the sequel, a distributed multi-agent DDQN algorithm to resolve the MOO problem (9) with the objective (10).

### 5.2. Distributed Multi-Agent DDQN Algorithm

In Section 3, the FP-based algorithm is introduced to represent a baseline to be obtained by an optimization-based algorithm. Given its merits on the centralized process, a distributed approach with lower time complexity is still considered better if each BS can independently determine its BP and PR with only limited information shared among their neighbors.

**Figure 2:**
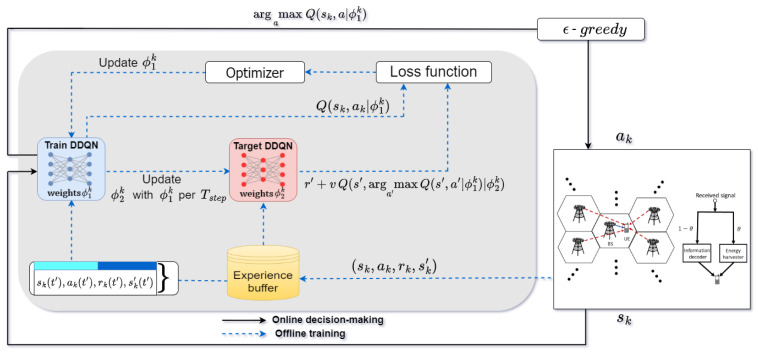
Structure of the proposed distributed DDQN algorithm in the multi-agent system.

To this end, the proposed DDQN algorithm is conducted to follow the concept of DTDE as shown in Figure 2, wherein each agent *k* takes its action $a_{k}$ based on its current state $s_{k}$ obtained from the information exchanged among its neighbors, representing the concept of *distributed executing* (DE). In addition, each agent *k* trains its own DNNs, $Q_{train}$ and $Q_{target}$, by using the experiences $\langle s_{k},a_{k},r_{k},s_{k}^{\prime }\rangle$ stored in its replay buffer $D_{k}$, representing *distributed training* (DT) in this algorithm. Specifically, the main MDP components for the proposed DDQN algorithm are summarized as follows:

(1) Action: In this algorithm, each action of agent *k* or $a_{k}$ is composed of BP $\left\{\omega_{k}\right\}$ and PR $\left\{\theta_{k}\right\}$. As the action space of value-based DRL algorithm must be finite, the feasible actions should be taken from a set of discrete values of $\left\{\omega_{k}\right\}$ and $\left\{\theta_{k}\right\}$, respectively. Here, as each BP is a complex vector, it should be discretized with real values. To this end, it is first decomposed into two parts as
  (33) $$\omega_{k}=\sqrt{P_{k}}{\bar{\omega }}_{k}$$
  wherein the first part, $P_{k}=\left|\right|\omega_{k}{\left|\right|}^{2}$, is the transmit power of BS *k*, and the second part, ${\bar{\omega }}_{k}$, represents the beam direction of BS *k*. On the one hand, the transmit power can be discretized linearly to constitute a set of values, such as $\left\{P_{min},P_{min}+\frac{P_{max}-P_{min}}{N_{p}-1},P_{min}+\frac{2(P_{max}-P_{min})}{N_{p}-1},\ldots ,P_{max}\right\}$ of $N_{p}$ equal-spacing values.
  On the other hand, ${\bar{\omega }}_{k}$ could be discretized by using a codebook $C=\left\{c_{0},\ldots ,c_{N_{code}-1}\right\}$ composed of $N_{code}$ code vectors $c_{k}\in C^{N_{t}\times 1}$, each specifying a beam direction in [0,2π). Providing a sufficient number of code $N_{code}\geq N_{t}$ to be adopted and a number of *S* available phase values for each antenna element, we can consider a codebook matrix C similar to that in [47]. Specifically, for the $n_{t}$-th antenna element in the *q*-th code, its value can be given by
  (34) $$C\left[n_{t},q\right]=\frac{\exp(j\frac{2\pi }{S}\left\lfloor \frac{n_{t}\;\mathrm{mod}\;\left(q+\frac{N_{code}}{2},N_{code}\right)}{N_{node}/S}\right\rfloor )}{\sqrt{N_{t}}}$$
  Apart from BP, we can similarly discretize each PR $\theta_{k}$ into $N_{eh}$ levels with a set $E=\left\{0,\frac{1}{N_{eh}-1},\frac{2}{N_{eh}-1},\ldots ,1\right\}$, representing its values to be selected. Finally, by taking all the discrete-value sets into account, we have the action space for each agent as
  (35) $$A=\left\{(p,c,e)|p\in P,c\in C,e\in E\right\}$$
  from which an agent *k* can choose its action $a_{k}\left(t\right)$ at time *t*.
(2) Reward: Apart from the above to select PR within [0,1] from E to comply with the feasible PR constraint, for the MOO problem, which is also required to meet the transmit power constraint, we conduct a dual form of this optimization by conceptually lifting the power constraint as the penalty term added in the objective to represent a reward to be obtained by the distributed multi-agent DDQN algorithm. Specifically, the reward function is denoted by
  (36) $$r=W\frac{C^{d}\left(\Omega ,\theta \right)}{\bar{C^{d}}}+\left(1-W\right)\frac{E^{h}\left(\Omega ,\theta \right)}{\bar{E^{h}}}-W_{c}P_{sum}$$
  where $W_{c}$ is the penalty weight, and $P_{sum}=\sum_{\forall i}\left|\right|\omega_{i}{\left|\right|}^{2}$ is the total transmit power consumption in the network. Given this, the reward of agent *k* at time *t* can be denoted by $r_{k}\left(t\right)=W\frac{C_{k}^{d}\left(t-1\right)}{\bar{C^{d}}}+\left(1-W\right)\frac{E_{k}^{h}\left(t-1\right)}{\bar{E^{h}}}-W_{c}P_{sum}\left(t-1\right)$.
(3) State: Conventionally, a state in MDP for RL-based algorithms is designed to represent the environmental information perceived by an agent. Given the same aim to represent as much available information as possible in the environment, the different problems involved, however, could realize their state spaces differently in the different related works, such as [39,40,48]. Here, to construct a state for this algorithm, an agent or BS *k* at time *t* will provide its local information about the direct link *k* at the previous time slot t−1 to its interferers $j\in I_{k}\left(t\right),\forall j$, including (1) the interference power received from *j*, $|h_{k,j}^{†}\left(t-1\right)\omega_{j}{\left(t-1\right)|}^{2}$; (2) the interference-plus-noise power, $\sum_{l\neq k}{\left|h_{k,l}^{†}\left(t-1\right)\omega_{l}\left(t-1\right)\right|}^{2}+\sigma^{2}$; (3) the achievable data rate, $C_{k}^{d}\left(t-1\right)$; and (4) the channel gain, $h_{k,k}^{†}\left(t\right){\bar{\omega }}_{k}\left(t-1\right)$. At the same time, it will also send the information to its interfered neighbors $i\in O_{k}\left(t\right),\forall i$, including the index $\ell_{k}\left(t-1\right)$ for the beam direction ${\bar{\omega }}_{k}\left(t-1\right)$ adopted and the achievable data rate $C_{k}^{d}\left(t-1\right)$.

In parallel, each interferer $j\in I_{k}\left(t\right)$ will send the index $\ell_{j}\left(t-1\right)$ for the beam direction ${\bar{\omega }}_{j}\left(t-1\right)$ and the achievable data rate $C_{j}^{d}\left(t-1\right)$ to agent *k*. Similarly, each interfered neighbor $i\in O_{k}\left(t\right)$ will send its measurements to agent *k*, including (1) the interference power, $|h_{i,k}^{†}\left(t-1\right)\omega_{k}{\left(t-1\right)|}^{2}$; (2) the interference-plus-noise power, $\sum_{l\neq i}{\left|h_{i,l}^{†}\left(t-1\right)\omega_{l}\left(t-1\right)\right|}^{2}+\sigma^{2}$; (3) the achievable data rate, $C_{i}^{d}\left(t-1\right)$; and (4) the channel gain, $h_{i,i}^{†}\left(t-1\right){\bar{\omega }}_{i}\left(t-1\right)$.

Given this, each agent *k* includes the following as the local information of its state, denoted by $s_{k}^{l}\left(t\right)$, as

- the normalized identity of BS, $k/N_{b}^{l}$;
- the normalized channel gain, $\left(\right|h_{k,k}^{†}\left(t\right){\bar{\omega }}_{k}\left(t-1\right){|}^{2})/N_{c}^{l}$;
- the normalized interference-plus-noise power,
  $(\sum_{l\neq k}|h_{k,l}^{†}\left(t\right)\omega_{l}\left(t-1\right){|}^{2}+\sigma^{2})/N_{i}^{l}$;
- the normalized reward, $\left(W\frac{C_{k}^{d}\left(t-1\right)}{\bar{C^{d}}}+\left(1-W\right)\frac{E_{k}^{h}\left(t-1\right)}{\bar{E^{h}}}-W_{c}P_{sum}\left(t-1\right)\right)/N_{r}^{l}$,

where $N_{b}^{l},N_{c}^{l},N_{i}^{l}$, and $N_{r}^{l}$ denote the normalization factors corresponding to the above four items, respectively. These factors (as well as the others to be introduced) for state normalization actually play a key role on preprocessing the training sample sets to lead to a much easier and faster training process as noted in [49,50]. Apart from that, the state of agent *k* also includes a set of information from its interferers, denoted by $s_{k}^{i}\left(t\right)$. Specifically, for each interferer $j\in I_{k}\left(t\right)$, it involves

- the normalized identity of the interferer BS, $j/N_{b}^{i}$;
- the normalized beam direction index adopted by the interferer BS, $\ell_{j}\left(t-1\right)/N_{i}^{i}$;
- the normalized interference power, $\left(\right|h_{k,j}^{†}\left(t-1\right)\omega_{j}\left(t-1\right){|}^{2})/N_{c}^{i}$;
- the normalized utility, $\left(W\frac{C_{j}^{d}\left(t-1\right)}{\bar{C^{d}}}+\left(1-W\right)\frac{E_{j}^{h}\left(t-1\right)}{\bar{E^{h}}}\right)/N_{u}^{i}$,

where $N_{b}^{i},N_{i}^{i},N_{c}^{l}$, and $N_{u}^{l}$ denote the corresponding normalization factors. In addition, a set of information from the interfered neighbors, denoted by $s_{k}^{d}\left(t\right)$, is also included in the state to completely describe the interference-limited environment for the MISO transmission. Specifically, the information for each interfered neighbor $i\in O_{k}\left(t\right)$ is represented by

- the normalized channel gain, $\left(\right|h_{i,i}^{†}\left(t-1\right){\bar{\omega }}_{i}\left(t-1\right){|}^{2})/N_{c}^{n}$;
- the normalized utility, $\left(W\frac{C_{i}^{d}\left(t-1\right)}{\bar{C^{d}}}+\left(1-W\right)\frac{E_{i}^{h}\left(t-1\right)}{\bar{E^{h}}}\right)/N_{u}^{n}$;
- the normalized SINR with respect to *k*,
  $\frac{|h_{i,k}^{†}\left(t-1\right)\omega_{k}{\left(t-1\right)|}^{2}}{\sum_{l\neq i}{\left|h_{i,l}^{†}\left(t-1\right)\omega_{l}\left(t-1\right)\right|}^{2}+\sigma^{2}}/N_{s}^{n}$;
- the normalized totally-received power,
  $\left(\sum_{\forall l}{\left|h_{i,l}^{†}\left(t-1\right)\omega_{l}\left(t-1\right)\right|}^{2}+\sigma^{2}\right)/N_{e}^{n}$,

where $N_{c}^{n},N_{u}^{n},N_{s}^{n}$, and $N_{e}^{n}$ are the normalization factors for the above four items, respectively. Note that, if agent *k* is not active in tim t−1, the numerator $|h_{i,k}^{†}\left(t-1\right)\omega_{k}{\left(t-1\right)|}^{2}$ as well as the whole SINR shown in the above are zero and will be excluded from the total received power as well.

Concatenating all three parts, we now have the state $s_{k}\left(t\right)$ = $\left\{s_{k}^{l}\left(t\right),s_{k}^{i}\left(t\right),s_{k}^{d}\left(t\right)\right\}$ for each agent *k*. Here, $|s_{k}\left|=\right|s_{k}^{l}\left|+\right|s_{k}^{i}\left|+\right|s_{k}^{d}|=4+4U+4U$ is the state size for each agent *k* to include the information from its *U* neighbors. Given this, the system state at time *t* can be denoted by $\left\{s_{1}\left(t\right),s_{2}\left(t\right),\ldots ,s_{L}\left(t\right)\right\}$. Then, following the principle of MDP, each agent *k* at time *t* will observe its own state $s_{k}\left(t\right)$ and choose its action $a_{k}\left(t\right)$ with the transition probability $P_{s_{k},s_{k}^{\prime }}^{a_{k}}$ determined by its DNN to move to the next state $s_{k}^{\prime }$.

(4) Selection policy and experience replay: Apart from MDP, the DDQN algorithm also adopts the same mechanisms usually found in DQN, such as ϵ-greedy selection policy and experience replay. First, by using the ϵ-greedy selection policy, each agent can explore the environment with the probability ϵ and can exploit with the probability 1−ϵ, where ϵ is a hyperparameter for the trade-off between exploration and exploitation and decays with a rate of $\lambda_{\epsilon }$ to its minimum value $\epsilon_{min}$, similar to that in [51]. Further, by means of experience replay, each agent *k* can store its transactions $\left(s_{k}\left(t\right),a_{k}\left(t\right),r_{k}\left(t\right),s_{k}^{\prime }\right)$ in a buffer memory $D_{k}$, and then randomly sample $D_{k}$ to construct a mini-batch for training its DNNs through, e.g., a stochastic gradient descent (SGD) algorithm to update the weights $\phi_{1}$ and $\phi_{2}$ for $Q_{train}$ and $Q_{target}$, respectively. As a summary, the proposed multi-agent DDQN algorithm is is shown in Algorithm 2 for reference.

**Algorithm 2** Multi-agent DDQN algorithm.
1:(Input) Simulated SWIPT MISO network and hyperparameters for the DDQN algorithm;2:(Output) Learned DDQN to decide $P_{k},{\bar{\omega }}_{k},\theta_{k},\forall k$, for MOO in (9) with objective in (10);3:Initialize a pair of $Q_{train}$ and $Q_{target}$ with $\phi_{1}^{k}$ and $\phi_{2}^{k}$ for each agent/BS $k\in \left\{1,\ldots ,L\right\}$4:Initialize state $s_{k}\left(0\right)$, action $a_{k}\left(0\right)$ and replay buffer $D_{k}=\emptyset$ for each agent *k*;5:**for** each time slot *t* **do**6:   **for** each agent/BS *k* **do**7:     Observe current state $s_{k}\left(t\right)$ in time slot *t*;8:     generate a random number $n_{r}$;9:     **if** $n_{r}<\epsilon$ **then**10:        Randomly select $a_{k}\left(t\right)$ from the action space A;11:     **else**12:        Select $a_{k}\left(t\right)=\arg\max_{a\in A}Q\left(s_{k},a|\phi_{1}^{k}\right)$;13:     **end if**14:     Observe next state $s_{k}^{\prime }$, and obtain reward $r_{k}\left(t\right)$;15:     Store the new transition $\left(s_{k}\left(t\right),a_{k}\left(t\right),r_{k}\left(t\right),s_{k}^{\prime }\right)$ in $D_{k}$;16:     Randomly sample a mini-batch $(s_{k}\left(j\right),a_{k}\left(j\right)$$,r_{k}\left(j\right),s_{k}^{\prime }\left(j\right))$ with $j\in J\subset D_{k}$ for experience;17:     Compute the Q-value for DDQN with (32)18:     Perform SGD to minimize the loss in (31), finding the optimal weights $\phi_{1}^{k}$ and $\phi_{2}^{k}$ of agent *k*;19:     Update weight $\phi_{1}^{k}$ (for $Q_{train}$);20:     Update weight $\phi_{2}^{k}$ (for $Q_{target}$) with $\phi_{1}^{k}$ every $T_{step}$ time slots;21:   **end for**22:
**end for**



Now, to evaluate its time complexity, we can assume that the neural network involved has *J* fully connected layers at most, in which $n_{j}$ denotes the number of neural units at the *j* layer, and $n_{0}$ is the input state size, leading to the complexity $O(\sum_{j=0}^{j=J-1}n_{j}n_{j+1})$ for its operations as noted in [49]. In addition, the DDQN algorithm is assumed to have $T_{m}$ time slots to learn, and, in each time slot, there are *L* distributed agents/BSs to train their own neural networks. Given this, the total complexity would be $O(T_{m}L\sum_{j=0}^{j=J-1}n_{j}n_{j+1})$.

Apart from the time complexity, each agent or BS requires at most four *U* messages from its neighbors with the limited channel information exchange. Otherwise, if a centralized approach in convention is adopted, the signal overhead would include the collection of $L^{2}$$N_{t}$-dimension complex vectors. In general, the number of neighbors for an agent or BS (i.e., *U*) is much less than the number of cells or BSs (i.e., *L*); thus, our approach can pay a lower signal overhead than can the centralized counterpart.

## 6. Numerical Experiments

In this section, we conduct simulation experiments to evaluate the proposed EA-aided FP algorithm (denoted by “FP”) and distributed multi-agent DDQN algorithm (denoted by “dis-DDQN”). To validate the proposed algorithms, we include a greedy-based algorithm and a random-based algorithm (denoted by “greedy” and “random”, respectively) as the comparison baselines. In addition, to verify the effectiveness of the DDQN algorithm based on DTDE, we introduce a CTDE variant (denoted by “glo-DDQN”), which uses the global state s = $\left\{s_{1},s_{2},\ldots ,s_{L}\right\}$ introduced in Section 5.2, to be the state for training each BS *k* instead of using only its local state $s_{k}$. Furthermore, to show the effectiveness of distributed computing, we also compare the Advantage Actor Critic (denoted by “A2C”) algorithm, which represents the state-of-the-art centralized RL algorithm to resolve this problem.

### 6.1. Simulation Setup

**Figure 3:**
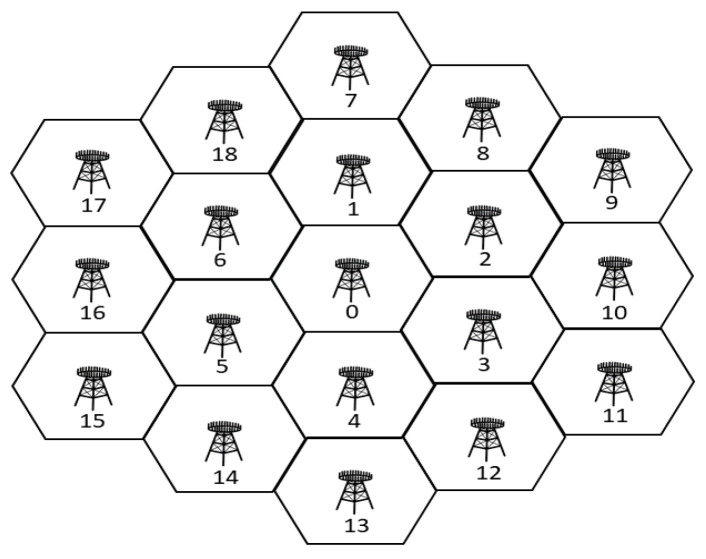
Simulation topology.

With the network and channel models introduced in Section 2, we set a simulation environment with 19 hexagonal cells with BS 0 located at the center, BSs 1–6 located in the first tier, and BSs 7–18 located in the second tier as shown in Figure 3, similar to the environment in [40]. However, unlike the previous, the cell radius was limited to 20 m for SWIPT to resemble that in a small cell, wherein the harvested energy would be significant enough in addition to the data transmitted.

Each UE is randomly located in each cell, and the path loss between BS *k* and UE *j* is similarly given by $\beta_{j,k}=120.9+37.6\log_{10}d_{j,k}$ dB, where the distance between them, $d_{j,k}$, is denoted in kilometers. Apart from the path loss, the signal was also generated with the log-normal shadowing effect, which had a standard deviation of 8 dB and AWGN noise power of −114 dBm. In addition, the number of multi-path was set to 4, and the difference between the maximum angle and the minimum angle, i.e., the angular spread, was $3^{\circ }$. Further, as UEs are located with random positions initially, the azimuth angle of UE to its BS serves as the direction of departure (DoD) of the wireless channel.

Apart from that, each channel had a time slot duration of 20 ms and a correlation coefficient of 0.64 for the successive time slots. As a summary, the important radio parameters with respect to the environment are tabulated in Table 1, and the import parameters and hyperparameters for DDQN are summarized in Table 2. Finally, along with W=0.5 for fairly weighting SE and EH in the first set of experiments and $W_{c}$ = $10^{-4}$ for the penalty of power consumption, the DDQN algorithms were conducted by a DNN with two hidden layers composed of 128 and 64 neurons, respectively.

In the parametric analysis, we first conducted different experiments to find the most suitable parameters for the multi-agent DDQN algorithm to be compared in the following, including the number of transmit power levels ($N_{p}$), the number of beam directions ($N_{code}$), and the number of power splitting ratios ($N_{eh}$). After that, we compared the proposed algorithms with the other schemes, and the results obtained confirm our proposal to outperform these benchmark schemes in terms of the utility U(Ω,θ), data rate $C^{d}\left(\Omega ,\theta \right)=\sum_{\forall i}C_{i}^{d}$, and harvested energy $E^{h}\left(\Omega ,\theta \right)=\sum_{\forall i}E_{i}^{h}$.

### 6.2. Parametric Analysis

#### 6.2.1. The Number of Power Levels

**Figure 4:**
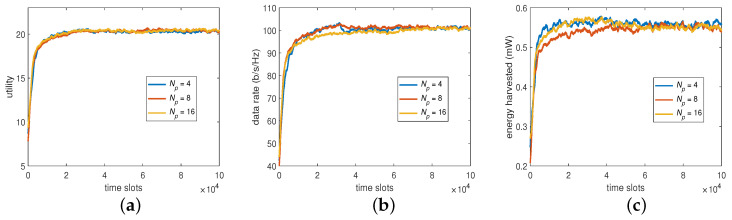
System performance by varying $N_{p}$: (**a**) utility, (**b**) data rate, and (**c**) harvested energy.

As shown in (33), there are two parts to constitute a BP. With respect to the first part of BP, transmit power, we set the transmit power to have 4, 8, and 16 levels of value for the Q learning to see its impact on the system performance. The results are summarized in Figure 4, showing that the different numbers of power levels $N_{p}$ provided similar utilities, data rates, and harvested energies. It implies that the algorithm may not, in this case, find the optimum represented through the values shown in these power sets even if $N_{p}$ and the overall state space increase. Thus, $N_{p}=4$ is considered sufficient in the sequel as it pays the lowest overhead for the algorithm to converge.

#### 6.2.2. The Number of Beam Directions

**Figure 5:**
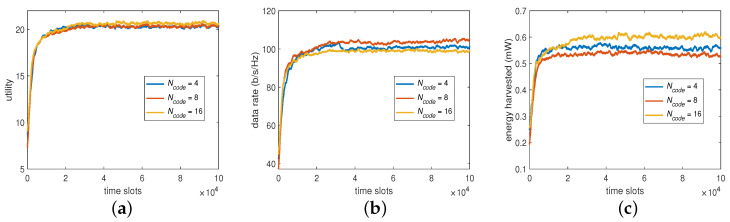
System performance by varying $N_{code}$: (**a**) utility, (**b**) data rate, and (**c**) harvested energy.

For the second part of BP, the beam direction, we set the codebook to have 4, 8, and 16 vectors or directions, respectively, to see its impact on the system performance. The results are now summarized in Figure 5, showing that $N_{code}=8$ could produce a higher data rate to compensate for a lower harvested energy and that $N_{code}=16$ could obtain a higher harvested energy to compensate for a lower data rate when compared with that of $N_{code}=4$. However, the trend is still the same in that increasing $N_{code}$ would provide similar utility as that on $N_{p}$. This suggests that, despite the slight trade-off between the data rate and harvested energy, $N_{code}=4$ would be sufficient for the algorithm to converge for the desired overall utility without further increasing its learning overhead.

#### 6.2.3. The Number of Power Splitting Ratios (PR)

**Figure 6:**
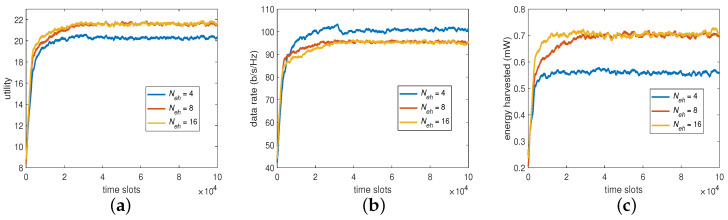
System performance by varying $N_{eh}$: (**a**) utility, (**b**) data rate, and (**c**) harvested energy.

Apart from BP, PR is another objective in our MOO problem. For the distributed DDQN algorithm, the number of PR level has the same importance as the former. To see its impact on the system performance, we provided a set of 4, 8, and 16 real values equally distributed between 0 and 1, for the experiments. As shown in Figure 6, $N_{eh}>4$ (i.e., $N_{eh}$ = 8 and 16) provided higher harvested energies and lower data rates, which eventually led to higher utilities compared with that of $N_{eh}=4$. However, to conduct the baseline for comparison without loss of generality, we adopted $N_{eh}=4$ as well as $N_{p}=N_{code}=4$, which exhibited the performance differences significantly enough for the DDQN algorithm in comparison and had a reasonable overall computational overhead.

Note that, as indicated in [52], when a multi-agent setting is modified by the actions of all agents, the environment becomes non-stationary from a single agent perspective, in which the effectiveness of most reinforcement-learning algorithms would not hold [53]. Thus, the performance of a multi-agent DRL algorithm does not guarantee an increase as the number of action increases through a trial-and-error mechanism in such environments [40] but could be explored by selecting suitable numbers of actions to constitute the action space as when performed for the proposed DDQN algorithm with the above experiments.

### 6.3. Performance Comparison

In this subsection, we exhibit the performance differences between the proposed algorithms and the other schemes. Specifically, based on the parametric analysis that we introduced, we set $N_{p}=N_{code}=N_{eh}=4$ for the multi-agent DDQN algorithm as well as a CTDE counterpart for a benchmark to be introduced in the following and $\ell_{m}=\ell_{\eta }=100$ for the FP algorithm. Then, we conducted a performance comparison between these algorithms and the other four benchmark schemes shown as follows:

- Global state information-based scheme: In principle, this scheme is the same as the distributed multi-agent DDQN algorithm. However, instead of adopting its own state $s_{k}$ only, each agent *k* adopts the full state information, i.e., $\left\{s_{1},s_{2},\ldots ,s_{L}\right\}$ for its own DDQN operations, based on the concept of centralized training distributed executing (CTDE). Clearly, collecting such information would require a centralized processor or a full information exchange mechanism to exist in the network and, thus, is denoted as “glo-DDQN” as noted at the beginning of this section.
- Single-agent DRL scheme: As a branch of machine learning, DRL is conventionally developed with a single agent operated centrally in a processor. Here, the state-of-the-art RL algorithm, Advantage Actor Critic, is adopted as a centralized DRL-based benchmark scheme for resolving the MOO problem and is simply denoted as “A2C”.
- Random-based scheme: As a baseline algorithm, the scheme leads each agent to randomly choose an action in each time slot and is denoted here as “random”.
- Greedy-based scheme: As another baseline algorithm, each agent in this scheme adopts the beam direction with the maximum channel gain and the maximum transmit power while randomly selecting its PR from the set of $N_{eh}$ elements for the DDQN. For easy reference, this scheme is denoted as “greedy” in the sequel.

**Figure 7:**
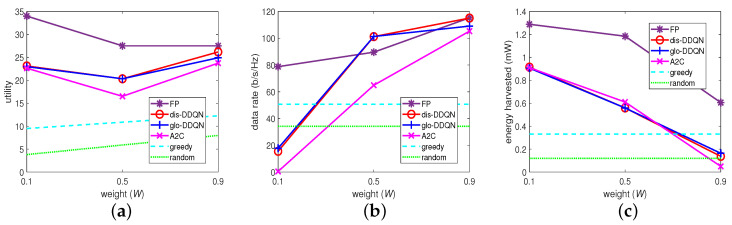
Comparison with different weights: (**a**) utility, (**b**) data rate, and (**c**) harvested energy.

For these algorithms, we set W=0.1, 0.5, and 0.9 in (10) to represent a “low”, “middle”, and “high” weight on the data rate (or a “high”, “middle”, and “low” weight on the harvested energy), and we examined the performance differences on these weights applied to these algorithms. Their results are summarized in Figure 7. Specifically, in Figure 7b,c, the random algorithm, which randomly chooses BP from the codebook despite *W* is shown to retain the same performance on these metrics, as expected. Similarly, given a non-zero *W*, each agent with the greedy algorithm chooses the best BP for its data rate despite the harvestable powers from the others, which are out of its control on BP, and this is also shown to remain the same on the two metrics when varying the weight.

Apart from these, the other algorithms exhibited similar trends, where increasing *W* increased the data rate and decreased the harvested energy, thus confirming the design aim of *W*. However, as the amount of the increased rate can be different from that of the decreased energy, their weighted sum or the resulting utility cannot be guaranteed to increase when *W* increases as shown in Figure 7a.

Given the similar trend, the FP-based algorithm (FP), which represents an optimization-based approach, is shown to provide the most effective solutions for the MOO problem, confirming our design aim. As shown in Figure 7a as well, the distributed multi-agent DDQN algorithm (dis-DDQN) has its overall utility under that of FP but outperforms the other schemes in comparison through the following viewpoints.

First, with respect to its variant (glo-DDQN), it can be observed that both algorithms (dis-DDQN and glo-DDQN) converge to similar results, and glo-DDQN can barely obtain a higher utility. The latter is possible because equipped with the global state information, each agent may need even more time to learn the strategy approaching the optimal system performance. It implies further that, with a higher overhead for learning, the large system state caused by glo-DDQN may not lead to a better result, a faster converging speed, or both, in time.

**Figure 8:**
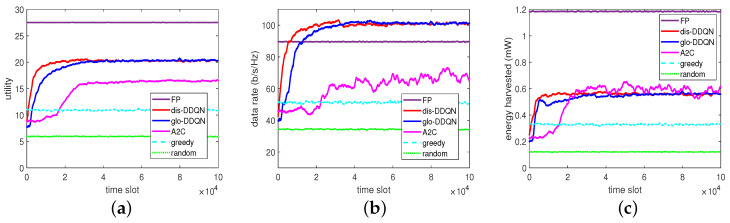
Comparison on convergence: (**a**) utility, (**b**) data rate, and (**c**) harvested energy.

From Figure 8, which exemplifies the converging progresses of these algorithms with W=0.5, it can be observed with more evidence that glo-DDQN actually converges more slowly than dis-DDQN in the time domain for all the metrics involved. Apart from the above, it can be also seen that the DDQN-based algorithms can obtain higher rates but provide relatively lower energies, which eventually leads to the overall utilities being lower than those obtained by the FP algorithm.

Second, with respect to A2C, which represents a state-of-the-art single-agent algorithm for the conventional environment to be evaluated centrally, it can be seen that such an algorithm may not work well in the distributed network with multiple BSs for a large state space, a large action space, or both. In other words, although A2C can handle the spaces involving both discrete and continuous variables (e.g., the beam direction is discretized while the transmit power, and the PR remains continuous in this case), its solution is not always efficient for the dynamic network environment. In contrast, by suitably discretizing the spaces involved, the distributed multi-agent DDQN (dis-DDQN) can be more easily handled by each agent to learn its strategy based on the limited discrete values in these spaces to approach the optimal solution.

Finally, in addition to the performance trends shown in the beginning, the greedy algorithm exhibits itself as a baseline scheme to provide a higher low-bound when no specific learning mechanism other than a greedy approach is adopted to resolve the MOO problem, and the random algorithm is shown to provide a lower low-bound on the performance if only randomly choosing an action is considered for solving this problem. As a summary, apart from the FP introduced, which represents an optimization-based approach to obtain outperforming solutions, the proposed DDQN algorithm (dis-DDQN) can also outperform the others in terms of the utility up to 1.23-, 1.87-, and 3.45-times larger than that of the A2C, greedy, and random algorithms, respectively, in comparison in the case of W=0.5.

**Figure 9:**
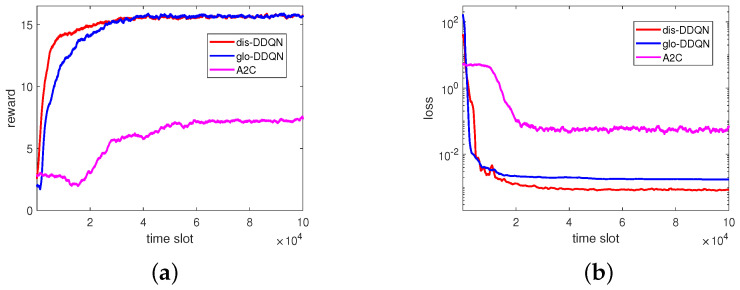
Reward and loss in the RL-based algorithms: (**a**) reward and (**b**) loss.

Apart from the above, we show, in Figure 9, the reward and loss for the RL-based algorithms in comparison. As can be easily seen, the reward increases and the loss decreases as time elapses, and dis-DDQN and glo-DDQN have higher rewards and lower losses compared to A2C, as expected. In particular, the lower losses found for the two DDQN algorithms suggest that the obtained models would perform better compared to A2C. To further validate the trained models from these RL-based algorithms, we prepared a set of 5000 test data by randomly generating channel fading conditions different from those of the training set.

**Figure 10:**
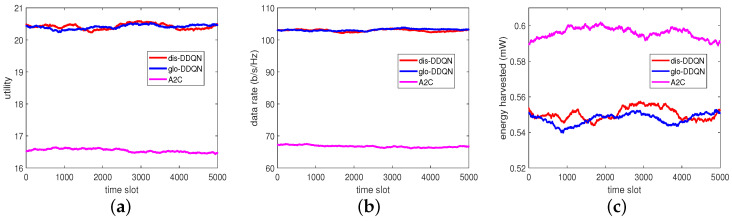
Test results of the RL-based algorithms: (**a**) utility, (**b**) data rate, and (**c**) harvested energy.

By reacting to the random data, each trained model can provide its own BPs and PRs, leading to the performance results summarized in Figure 10. From this figure, we can see that the test can consistently give outputs similar to those at the end of training, despite the different random unseen data for testing. This observation indicates that the trained models would have good generalization performance as expected.

## 7. Conclusions

In this work, a MOO problem was formulated that aims to obtain the optimal BP and PR concurrently for MISO downlink SWIPT-enabled wireless networks. For this problem, a weighted sum approach was conducted to make a trade-off between SE and EH in the Pareto-optimal sense. Given this, an EA-aided quadratic transform technique was proposed to conduct an FP-based algorithm that can obtain near-optimal solutions with the computationally-efficient iterative update procedure introduced. At the same time, a DTDE scheme was adopted to introduce a multi-agent DDQN algorithm that requires only partial observations of CSI for local computation in each agent to further reduce the communication overhead and the computational complexity.

With the simulated environment, our experimental results demonstrated that, among the benchmark schemes conducted, the introduced FP-based algorithm was the most effective approach for solving the MOO problem. Apart from the FP algorithm, the proposed multi-agent DDQN algorithm was also shown to outperform A2C, which represents the state-of-the-art single-agent DRL algorithm and the other baseline schemes while providing lower overhead and complexity compared with that of FP. This reveals the possibility that a programming-based method and a DRL-based algorithm can complement each other to solve various optimization problems in networking, and a joint design to take benefits from both will be our future work.

## Figures and Tables

**Table 1:** Radio parameters.

Parameter	Value
Number of neighboring cells (*U*)	5
Noise power ($\sigma^{2}$)	−114 dBm
Standard deviation	8 dB
Number of multi-paths	4
Time slot duration	20 ms
Angular spread	3°
Channel correlation coefficient	0.64
Cell radius	20 m
Maximum transmit power ($P_{max}$)	38 dBm
Minimum transmit power ($P_{min}$)	0
Number of transmit antennas in BS ($N_{t}$)	4
Number of transmit power levels ($N_{p}$)	4, 8, 16
Number of energy harvesting ratios ($N_{eh}$)	4, 8, 16
Number of beam directions ($N_{code}$)	4, 8, 16

**Table 2:** Parameters and hyperparameters for DDQN.

Parameter	Value
Learning rate	0.0005
Greedy exploration parameter (ϵ)	0.2
Exploration decay rate ($\lambda_{\epsilon }$)	0.0001
Minimum exploration rate ($\epsilon_{min}$)	0.01
Greedy decay rate	0.0001
Size of state for angent/BS *k* ($|s_{k}|$)	44
Size of action for angent/BS *k* ($|a_{k}|$)	64
Replay buffer size for angent/BS *k* ($|D_{k}|$)	500
Batch size for angent/BS *k*	32
Normalization factors for local BS ($N_{b}^{l},N_{c}^{l},N_{i}^{l},N_{r}^{l}$)	$\left(1,10^{-4},10^{-4},1\right)$
Normalization factors for interferer BS ($N_{b}^{i},N_{i}^{i},N_{c}^{i},N_{u}^{i}$)	$\left(18,1,10^{-4},10\right)$
Normalization factors for interfered BS ($N_{c}^{n},N_{u}^{n},N_{s}^{n},N_{e}^{n}$)	$\left(10^{-4},1,10,10^{-2}\right)$

## Data Availability

Not Applicable.

## Notations

Following the writing convention, vectors and matrices in this work are denoted by boldface lowercase and uppercase symbols (e.g., x and X), respectively. The Hermitian transposition and inverse are denoted by the superscripts ${\left(\cdot \right)}^{†}$ and ${\left(\cdot \right)}^{-1}$, respectively. In addition, |·| denotes the absolute value operator, and ||·|| is that for the Euclidean norm. Further, Re(·) is the operation to take the real part, and $\hat{I}$ denotes an identity matrix.
